# An Update on the Measurement of Motor Cerebellar Dysfunction in Multiple Sclerosis

**DOI:** 10.1007/s12311-022-01435-y

**Published:** 2022-06-27

**Authors:** Katherine Hope Kenyon, Frederique Boonstra, Gustavo Noffs, Helmut Butzkueven, Adam P. Vogel, Scott Kolbe, Anneke van der Walt

**Affiliations:** 1 Department of Neuroscience, Central Clinical School, Monash University, Melbourne, VIC Australia; 2 Centre for Neuroscience of Speech, University of Melbourne, Melbourne, VIC Australia; 3 Department of Neurology, Royal Melbourne Hospital, Melbourne, VIC Australia; 4 Redenlab Inc, Melbourne, VIC Australia; 5 Division of Translational Genomics of Neurodegenerative Diseases, Hertie Institute for Clinical Brain Research, University of Tübingen, Germany & Center for Neurology, University Hospital Tübingen, Tübingen, Germany; 6 The Bionics Institute, Melbourne, VIC Australia; 7 Department of Medicine and Radiology, University of Melbourne, Parkville, Australia

**Keywords:** Multiple sclerosis, Cerebellum, Neuroimaging, Acoustic speech analysis, Home-based monitoring

## Abstract

Multiple sclerosis (MS) is a progressive disease that often affects the cerebellum. It is characterised by demyelination, inflammation, and neurodegeneration within the central nervous system. Damage to the cerebellum in MS is associated with increased disability and decreased quality of life. Symptoms include gait and balance problems, motor speech disorder, upper limb dysfunction, and oculomotor difficulties. Monitoring symptoms is crucial for effective management of MS. A combination of clinical, neuroimaging, and task-based measures is generally used to diagnose and monitor MS. This paper reviews the present and new tools used by clinicians and researchers to assess cerebellar impairment in people with MS (pwMS). It also describes recent advances in digital and home-based monitoring for people with MS.

## Introduction

Multiple sclerosis (MS) is a debilitating disease of the central nervous system [1] and is one of the leading causes of disability in young and middle-aged adults [2]. The disease has been described since the 1800s, with fluctuating speech impairments, muscle weakness, pain, and vision impairment being among the symptoms mentioned in the earliest accounts [3–6]. The clinical presentation of MS is highly heterogeneous, which makes individual clinical outcomes difficult to predict. Possible symptom combinations at various severity levels differ between disease types and individual people with MS (pwMS) [7]. This is further impacted by other factors such as age at onset, mental and physical health, and socioeconomic status [8–10].

Nonetheless, symptoms can be linked back to specific regions of the central nervous system. Among regions of interest, the cerebellum plays a crucial role in sensory, motor, cognitive, and behavioural processes and is often impacted during MS by inflammatory demyelinating lesions [11]. Symptoms associated with cerebellar injury include ataxia, upper limb incoordination, dysarthria, and tremor [1]. Cerebellar symptoms are common in MS, with up to one third of pwMS experiencing these [12]. When present, cerebellar dysfunction contributes significantly to an increased rate of disability, reduced mobility, and impaired quality of life [11]. Cerebellar dysfunction experienced in the first 2 years after onset is related to a 20% increase in future overall disability [13]. There are several ways of diagnosing and monitoring cerebellar symptoms in MS. Here, we review and summarise the current methods for measuring cerebellar dysfunction in pwMS with a focus on emerging technologies including advanced neuroimaging, automated speech analysis, and home-based electronic testing.

## The Cerebellum

### Anatomy and Role

**Fig. 1:**
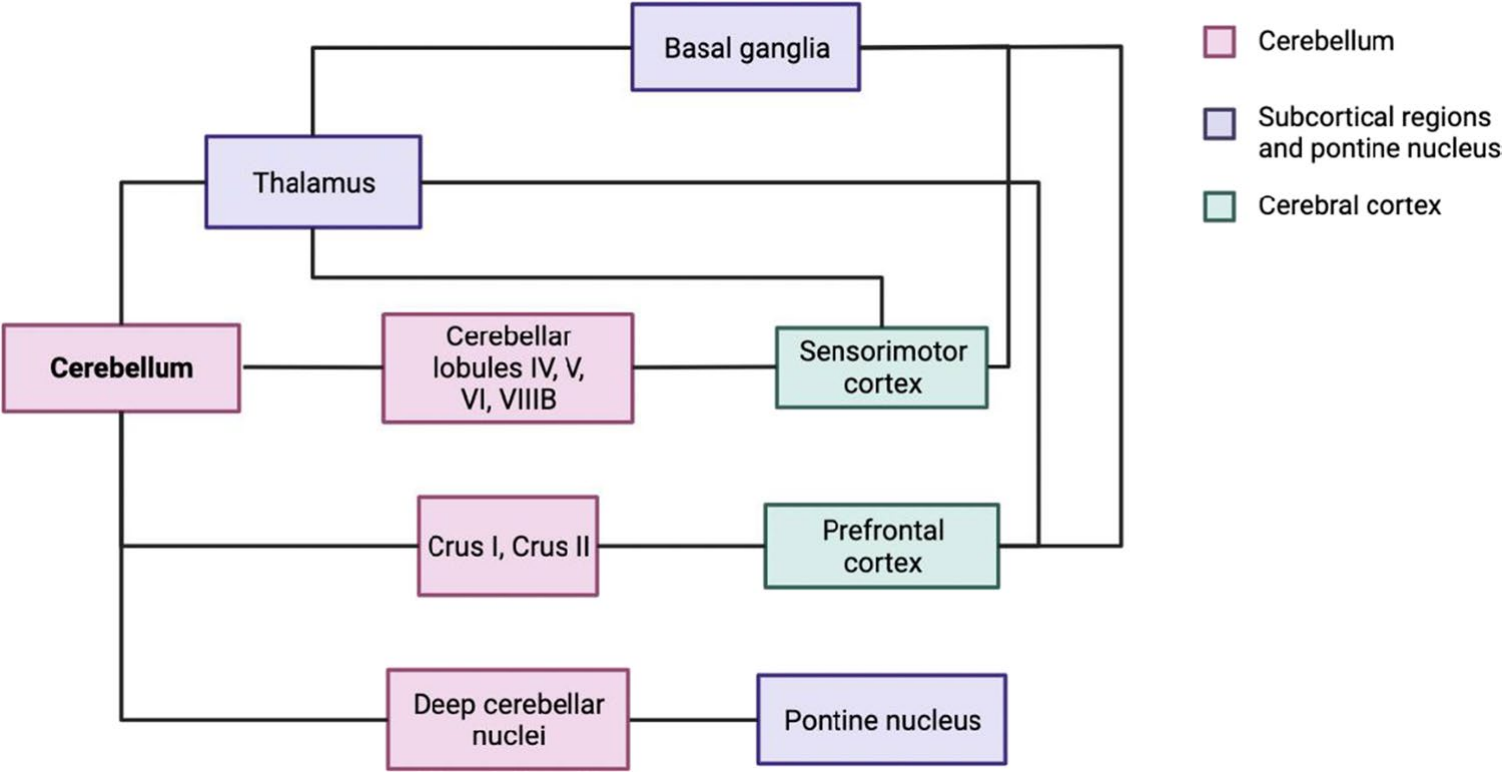
Motor networks involving the cerebellum. Created with BioRender.com

The cerebellum is neuronally dense and accumulates several functions within the central nervous system. Despite its small size relative to the cerebrum, the cerebellum contains over 100 billion neurons compared to just 86 billion in the cerebrum [11]. The cerebellum integrates multiple circuits throughout the brain and is involved in motor, cognitive, and emotional functions. It is connected to the parietal, somatosensory, visual, auditory, prefrontal, motor, and premotor areas within the cerebral cortex through parallel connectivity loops [14]. Functional connectivity (FC) studies show distinct neural networks comprising of the cerebellum and specific regions of the cerebrum. For example, the motor cortex is associated with lobules IV-VI and VIIIB of the cerebellum, whereas areas of the prefrontal cortex connect to Crus I and Crus II [14, 15]. Purkinje cells form the output system of the cerebellum to the rest of the CNS [16]. Purkinje cell activity is associated with motor learning, coordination, and control. Furthermore, they are crucial in integrating sensory and motor signals, thus controlling sensorimotor behaviours [17]. In addition, the cerebellum plays a vital role in motor learning and memory and is highly plastic, with many forms of neural plasticity being reported [11]. Droby and colleagues [18] found an increase in FC during an acute MS relapse associated with a new white matter (WM) lesion. They believe this to be indicative of recruiting intact regions of the brain to carry out tasks [18]. A further correlation between structural damage and increased FC backs up this conclusion. Rocca et al. [19] suggest an increase in FC is an adaptive response to damage to WM bundles. During a motor task using the right hand, their study found increased activity in the left supplementary motor area, left primary sensorimotor cortex (PSMC), and right cerebellum. Rocca and colleagues also found increased FC between the right PSMC and right cerebellum, which correlated with tissue damage in the dentatothalamic and corticospinal tracts [19]. The cerebellum may therefore have a specific role in mediating FC changes following structural damage related to MS [19, 20]. See Fig. 1 for the summarised cerebellar motor connectivity.

### Cerebellar Dysfunction in MS

Cerebellar impairment can start at any stage of the MS disease course [21]. Cerebellar pathology includes both grey matter (GM) and WM lesions, reduced Purkinje cell density, and neuronal loss [22]. Infratentorial lesions are associated with long-term disability [23], with the cerebellar peduncles being among the most impacted regions in terms of lesion density [24]. Recent studies have also shown that the pons and cerebellar peduncles specifically have higher lesion frequency than other areas in people with clinically isolated syndrome (CIS, the precursor to MS) [24, 25]. Autopsy research has shown an average of 38.7% of the cerebellar cortical area is affected by demyelination in pwMS, with the most severe cases reaching over 90% [26].

Cerebellar dysfunction can occur as part of an acute relapse or, perhaps more commonly, as a feature of progressive worsening in advanced MS [1]. In fact, the presence of cerebellar symptoms of MS is associated with an increased risk of developing a progressive disease course [27]. Lower cerebellar volume and higher T2 lesion load are associated with increased cognitive and motor difficulties and are correlated with higher clinical disability as measured by the Expanded Disability Status Scale (EDSS) [21]. T2 lesions in the middle and superior cerebellar peduncles are common in pwMS and are associated with disease severity and upper limb function [1, 28]. Furthermore, the cerebellar cortex is also affected by demyelination which increases in persons with progressive MS [26]. At the other end of the disease spectrum, decreases in cerebellar WM and total volume compared to controls have been described in early MS and in clinically isolated syndrome (CIS, the precursor to MS) [29].

Clinical cerebellar dysfunction (tremor, limb and gait ataxia, dysarthria, etc.) more often persist after a relapse than, for instance, sensory changes [21, 30] and can be challenging to manage, thus further adding to morbidity. Because of the organisation of the cerebellum and its different network connections, it is possible to identify location-specific deficits. For example, lesions to the midline area of the cerebellum cause dysfunction of simple motor tasks. Conversely, damage to the lateral cerebellum results in impairment of more complex motor tasks and cognitive deficits. Cognitive deficits include motor planning and language production [31–33]. Injury to the superior cerebellar peduncle, identified using diffusion tensor MRI-derived fractional anisotropy, is associated with reduced upper limb function and walking speed in pwMS [34]. Additionally, attention, verbal, and visual memory impairments correlate with reduced regional resting-state FC in cerebellar networks [35]. The dentate nucleus, a large cluster of neurons in the cerebellum, is involved in motor control, cognition, language, and sensory functions. It also connects to motor and cognitive association areas in the cerebral cortex [36]. In pwMS, researchers using light microscopy have found a significant reduction in afferent dentate synapses in areas both with and without demyelination in post-mortem cerebellar tissue [27]. They also observed atrophy and reduction of dentate neurons in pwMS, thus providing further information regarding cerebellar pathology in MS. Moreover, neuroimaging has shown altered dentate FC to frontal regions at rest in pwMS with an inverse correlation between FC and both T2 lesion volume and clinical impairment [37]. Additionally, any damage that disrupts communication between the cerebellum and higher-level cortical areas can contribute partly to cognitive impairment seen in pwMS [30, 38]. This manifests clinically as executive dysfunction and a decline in memory and language performance.

## Clinical Measures of Cerebellar Dysfunction

### Subjective Scoring Measures (Table 1)

**Table 1:** Clinical measures of cerebellar dysfunction in pwMS

Clinical score used	Author	Number of participants	Method/design	Findings
9HPT, EDSS	Goodkin, Hertsguard [130]	89	Compare the 9HPT and box-and-block test to EDSS to determine sensitivity	9HPT is sensitive to changes in functional status associated with upper limb dysfunction as measured by the EDSS
EDSS, KFSS	Noseworthy, Vandervoort [131]	168	Assess inter-rater variability in EDSS and KFSS in pwMS	Change in degree of disability associated with a 1 point change in EDSS score and a 2 point change in KFSS score
EDSS, KFSS, 9HPT	Cutter, Baier [132]	5,457 from 15 datasets	Assess EDSS, KFSS, and MSFC (including the 9HPT) over time	Significant correlation between the EDSS, 9HPT, and disease duration. A strong correlation was found between the 9HPT and cerebellar FSS
9HPT	Erasmus, Sarno [133]	482 (*N* = 240 pwMS, 140 controls)	Repeated measures design using clinical scales and kinematic and spectral analysis to determine level of ataxic symptoms	Able to distinguish between pwMS and controls and able to distinguish those with clinical cerebellar dysfunction
KFSS	Kalron and Givon [134]	289 (*N* = 147 with cerebellar scores)	Assess gait using pyramidal, sensory, and cerebellar scores	Pyramidal function plays the highest role in gait. No significant differences with added cerebellar dysfunction
ICARS, SARA	Salcı, Fil [41]	80	Assessed pwMS with ataxia using SARA and ICARS, correlated with EDSS and cerebellar KFSS	High inter-rater reliability, ICARS has sig correlations with EDSS and KFSS cerebellar scores, suggesting high validity
9HPT	Solaro, Cattaneo [135]	363	Determine correlation between 9HPT scores, EDSS scores and MS type using a cross-sectional study involving multiple MS centres	Floor and ceiling effects for mild and severe cases of MS. Higher EDSS and people with primary progressive MS showed more asymmetry in hand function
EDSS	Le, Malpas [13]	10,513	Data from MSBase registry. A mixed-effects model used to determine associations between early cerebellar presentations and EDSS scores	Cerebellar symptoms early on are associated with higher EDSS scores independent of pyramidal dysfunction They may be used as markers for disease progression

Disease severity and neurological impairment in MS are commonly defined using a standardised clinical assessment, the EDSS. This scale was designed to describe disease progression in pwMS and uses an ordinal scale from 0 (normal neurological status) to 10 (death due to MS) [39]. The associated subscores, or so-called Kurtzke functional system (KFS) scores, were designed to address different neurological areas of dysfunction, including the cerebellar and brain stem functional systems [40]. The KFS score for these two functional systems incorporates symptoms of ataxia, nystagmus, dysarthria, swallowing difficulties, and extraocular weakness [40]. Higher cerebellar KFS scores predict a shorter time to reach an EDSS score of 6, where one requires aid to walk 100 m [12]. The other available clinical scores, although often used in MS studies, were all developed with different diseases in mind. The international cooperative ataxia rating scale (ICARS) measures ataxia-related symptoms on four subscales: posture and gait disturbances, speech disorders, kinetic functions, and oculomotor disorders [41]. The ICARS has 19 items used to assess ataxia severity and is scored out of 100 [42]. A third clinical rating system specific to cerebellar ataxia is the scale for the assessment and rating of ataxia (SARA). This measure is scored out of 40 and comprises eight different items that evaluate gait, speech, truncal postural, and limb kinetic function [43] The SARA has been validated in MS and demonstrates high test–retest reliability and internal consistency for pwMS with ataxia [41]. In addition, the score increases as cerebellar ataxia symptoms worsen, making it a valid measure of cerebellar ataxia [43].

### Objective Measures

The nine-hole peg test (9HPT) assesses upper limb dexterity in pwMS [44]. It accurately distinguishes between controls and pwMS with different levels of impairment. The 9HPT is a common part of the multiple sclerosis functional composite (MSFC) alongside walking, visual, and cognition tasks [45].

We have summarised the clinical measures of cerebellar dysfunction in pwMS in Table 1.

### Kinematic Analysis of Gait and Balance

Gait and balance dysfunctions are common in MS and correlate with cerebellar damage [46]. Subtle changes to gait and balance are also precursors to a more severe loss of mobility in pwMS [47]. Therefore, early detection of subtle gait changes can be used to predict mobility loss later in the disease course. There are several ways to measure gait and balance in pwMS, including wearable and non-wearable options. Non-wearable measures such as the instrumented treadmill and the butterfly diagram are more accurate and reliable but tend to require specialised equipment while also being inconvenient [47, 48]. On the other hand, wearable systems, although perhaps providing less detailed information, can be used in community settings and at home and give real-time feedback to patients. One example is the use of inertial measurement units (IMUs). IMUs are small, light integrated systems that measure the linear and angular motion of the wearer. These systems can be attached anywhere on the body but are commonly positioned on the lower back, sternum, calf, wrist, or ankle [49]. IMU harmonic ratios in people with cerebellar ataxia — a common symptom of MS — correlate with ataxia severity and gait features such as stance, swing, and double support duration [50]. This has also been found in pwMS with gait dysfunction where IMUs can quantify speed, step length, and step time. These measures correlate with EDSS scores [49]. IMUs can also be used to measure postural sway — an aspect of balance control — in pwMS [51]. Increased standing postural sway is associated with higher EDSS scores, specifically higher cerebellar KFSS subsystem scores [52]. New technology now also allows for inertial/passive data collection on smartphones and watches, making them more accessible and user-friendly for patients [47]. Inertial sensors show that postural sway deficits are associated with reduced WM integrity in the superior and inferior cerebellar peduncles in pwMS [53]. There are also simple standing and walking assessment options, including the 2- and 6-min walking tests that are frequently included in clinical trials. However, these tests can be limited by inter-test variability and lack of sensitivity to subtle changes in gait.

### Limitations of Current Clinical Assessments

Clinical assessments, especially the EDSS and its subscores, remain the gold standard for monitoring MS disease status and progression. However, there are several limitations of current clinical assessments of cerebellar dysfunction. Firstly, the ICARS and SARA are not MS-specific [41], and their scores are primarily related to the level of ataxic symptoms such as posture, gait, and limb kinetic function. These items make up a possible 86 of the 100 points in the ICARS, and 30 out of 40 points of the SARA [41]. In addition, the ICARS is not always sensitive to change over time, especially with long disease durations [45, 54]. The cerebellar KFSS also focuses on gait ataxia and an increase in score requires a higher level of interference with daily functioning. While ataxia is an important symptom of cerebellar dysfunction to monitor, it is important not to underestimate the impact of other cerebellar symptoms such as tremor and dysarthria on quality of life and patient function. Furthermore, the EDSS is known to have limited inter-rater reliability [45], while the 9HPT has practice effects to consider when used alone or as part of the MSFC [45]. The 9HPT also solely assesses upper limb function and does not measure other cerebellar features such as gait [55]. Moreover, walking tests of gait have high variability depending on the precision and accuracy of measurement devices [56] and variation in task protocol [57, 58]. There is also little research on whether IMUs can indicate changes in MS disease severity over time [49]. The evidence thus far underscores that no single clinical measure provides enough information on both cerebellar function and overall MS disease-related impairments. It is, therefore, crucial to extend disease diagnosis and monitoring into paraclinical measures.

## Neuroimaging Measures of Cerebellar Dysfunction

Magnetic resonance imaging (MRI) has a well-established role in research and clinical practice in MS. MRI is sensitive to different pathological substrates of MS including inflammatory demyelination and neuro-axonal loss [22]. More advanced MRI methods can derive quantitative objective measures that provide pathophysiological insights into MS pathogenesis. The cerebellum, as part of the infratentorial regions of the brain, is commonly assessed for the dissemination in space criterion of MS diagnosis [59]. MRI can be used to measure structural abnormalities and changes in cerebellar volume. Furthermore, MRI can be used to monitor connectivity between the cerebellum and cerebrum and changes in metabolism and blood flow. This allows us to monitor cerebellar function.

### Lesion and Volumetric MRI

Demyelinating lesions and brain atrophy in MS are universal features of the disease across every stage of evolution, and the cerebellum is no exception. Cerebellar WM volume decreases in pwMS compared to healthy controls [30, 34, 60], whereas T1-weighted MRI can differentiate between groups of people with RRMS, SPMS, CIS, and healthy controls through analysis of mean cerebellar GM volume [60]. Similarly, cerebellar lesions are frequently detected by MRI [22]. Cerebellar leukocortical or WM lesions correlate with cerebellar volume loss and dysfunction in pwMS [61]. Additionally, overall increased T2-weighted cerebellar lesion volume and lower anterior cerebellar volume are associated with slower performance on the 9HPT [21]. There is higher volume and frequency of T2 lesions in the middle and superior cerebellar peduncles in pwMS with cerebellar and brainstem symptoms [62]. This damage is more precisely related to walking impairments in pwMS than measures of lesion volume or cerebellar atrophy [62]. Altered attention, verbal fluency, and motor performance are associated with total lesion load and mean lesion volume [63]. While this correlation is visible at 3 T, 7 T scanner findings showed significantly higher lesion load than lower level scanners [63]. A 2020 study showed that, compared to 3 T, 7 T scanners have up to 134% higher sensitivity for lesion detection. This led to better discrimination between cortical and WM lesions, and between leukocortical and WM lesions within the cerebellum in pwMS [64]. However, while lesion characterisation and volumetric MRI measures at all field strengths are useful for assessing structural changes associated with MS disease activity, they do not address any changes in FC or activity. Furthermore, they do not address microstructural changes in pwMS.

### Advanced Neuroimaging Techniques

#### Diffusion-Weighted MRI

Diffusion MRI tracks the motion (i.e., diffusion) of water molecules in the brain [65]. Diffusion occurs with greater ease along tracts (parallel to axons) and less so when perpendicular to microstructural barriers (e.g., cell walls, extracellular sheets). Abnormalities in diffusion are often found in lesions and normal-appearing WM (NAWM) in pwMS [66, 67]. Diffusion abnormalities in the middle and superior cerebellar peduncles correlate with T2 lesion load in these regions, as well as with whole-brain T2 lesion load and cerebellar GM volume [62]. Research has found significant differences in diffusion in MS lesions when compared to contralateral and healthy tissue in the cerebrum [66]. The residual signal fraction, a measure of the volume fraction of axons, was also able to distinguish between NAWM and lesions in pwMS [66]. Additionally, normal-appearing GM (NAGM) in pwMS has microstructural damage, the extent of which correlates with the number of lesions throughout the brain and with cognitive impairment [67]. NAGM mean diffusivity and fractional anisotropy also positively correlate with EDSS scores [67]. Diffusion imaging of the cerebellum can accurately group mean differences between pwMS and controls or between pwMS with and without clinical impairment measured by the EDSS, cerebellar, and brain stem FSS [62]. Cerebellar diffusion metrics such as fractional anisotropy and radial diffusivity are correlated with EDSS scores in pwMS [62, 68]. Thus, diffusion metrics are associated with how microstructures are arranged in the CNS.

#### Functional MRI

Changes in functional activity and connectivity throughout the brain are some of the neurophysiological characteristics of MS [68]. Functional MRI (fMRI) uses blood oxygen level-dependent (BOLD) contrasts to track blood flow associated with neural activity [65]. BOLD contrasts show differences between pwMS and controls in connectivity, level of activation, and areas of activation in the brain [65]. fMRI can therefore demonstrate various functional abnormalities in the brains of pwMS that can be maladaptive, for example, reduced activation in the sensorimotor network. Adaptive function can also be evidenced by fMRI, such as increased activation and recruitment of additional brain areas during cognitive tasks [69]. Longitudinal research has shown that functional abnormalities vary, both during relapse and during periods of clinical stability [69]. Functional abnormalities strongly correlate both with disease severity and structural MRI findings [70].

fMRI studies involving the cerebellum can provide unique insights into its complex connections and functions. One study found a reduction in regional homogeneity of BOLD signal changes in pwMS within the left cerebellar hemisphere. In Crus I, Crus II, and dentate nucleus specifically, abnormal regional homogeneity also correlates with clinical disability [71]. Abnormal FC in the cerebellum identified through fMRI, both during active tasks and at resting-state (RS), has been linked to more severe disability and a higher number of inflammatory lesions [11]. RS fMRI has an extra advantage in MS research in that it allows us to perform functional imaging studies with pwMS who struggle completing tasks [72]. Higher cerebellar RS FC correlates with less severe disability in pwMS, which suggests an adaptive role for preserving clinical function [72]. Increased RS FC in the dentate nucleus is similarly linked to better motor performance, shorter disease duration and lower T2 lesion volume [73]. However, reduced RS FC in the dentate nucleus is associated with longer disease duration, cognitive impairment and higher T2 lesion volume in paediatric MS cases, possibly reflecting a loss of adaptive neuroplasticity [73].

Using task-based fMRI, motor dysfunction such as tremor has also been linked to cerebellar damage in pwMS [74, 75]. Additionally, fMRI has been used alongside speech analysis to identify the cerebellar function in the motor control of speech production in people with dysarthria [76]. Ackermann and Hertrichh’s 2000 study found preliminary evidence that cerebellar activation occurs at or above a speech tempo of 3 Hz during a syllable repetition task, which suggests that the cerebellum plays a role in the speed of articulatory movements after a certain base-level [76]. Like the increased sensitivity in structural imaging, ultra-high field fMRI can detect more minute changes in cerebellar functioning. While subtle impairments may not be picked up in clinical tasks, 7 T fMRI is able to detect changes in brain activity associated with upper and lower limb movement changes in minimally disabled pwMS (EDSS score < 4, pyramidal and cerebellar KFSS scores ≤ 2) [75].

### Limitations of Neuroimaging Measures

In comparison to the cerebrum, the cerebellum has been less studied in MS. This is partly explained by contrast and resolution limitations of clinical MRI [77]. Additionally, optimal imaging of the cerebrum often takes precedence over and consequently limits that of the cerebellum in the clinical management of MS [78]. It is therefore not surprising that there are only a limited number of automated cerebellar segmentation algorithms available [77].

While sensitive to various pathological processes associated with MS, MRI cannot identify all underlying disease pathology [79]. For example, NAWM in T1- and T2-weighted images may still have widespread histopathological abnormalities [79]. Secondly, clinical MRI alone does not adequately explain the gradual disease progression typical in SPMS [79, 80].

Biological confounds can significantly influence both clinical MRI and fMRI. Volumetric MRI can be impacted by natural atrophy occurring with aging, level of hydration, and lifestyle factors including smoking and alcohol consumption [81]. Measurement inaccuracy in volumetric MRI therefore limits its clinical use for short-term assessment in individual pwMS. Additionally, biological artefacts such as cardiac and breathing cycles are more pronounced in cerebellar fMRI than cerebral fMRI. There are very few longitudinal fMRI studies in MS and even fewer that focus on the cerebellum [11, 82]. Furthermore, uncertainty remains about what type of neural activity is reflected in the cerebellar BOLD signal [83]. Consequently, additional research in this area is crucial for developing methods for monitoring cerebellar injury over time in pwMS.

It is also important to note that neuroimaging is less accessible than, for instance, clinical cerebellar monitoring. Imaging equipment is not always available, and when it is, it can be costly [84]. New MRI sequences can increase the scan-time of MRI, leading to patient discomfort that is further increased in those with more advanced disability [85].

## Speech Measures of Cerebellar Dysfunction

**Fig. 2:**
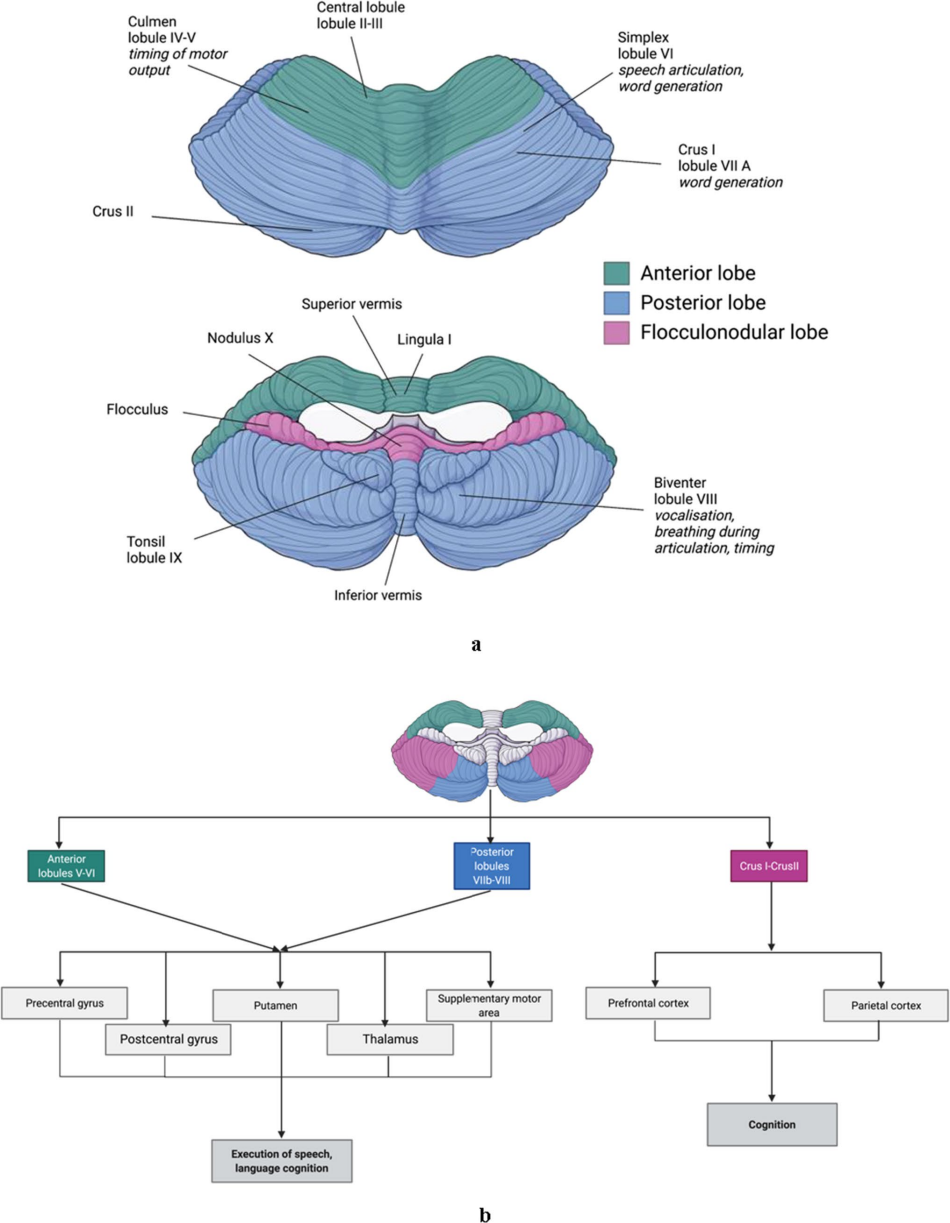
**a** Regions of the cerebellum and their motor speech functions. Created with BioRender.com. **b** Cerebellar connectivity networks and their role in speech production. Created with BioRender.com

Speech disorders are relatively common in pwMS, with 40–50% of pwMS experiencing difficulties with motor speech production (i.e., dysarthria) [86]. The resulting difficulties in communication often impact self-image, cause feelings of isolation, and decreased quality of life in pwMS [87]. White and GM loss, and damage to the bilateral corticobulbar tracts, cerebellum and midbrain are linked to dysarthria in MS [88, 89]. Furthermore, increased severity and frequency of dysarthric symptoms are associated with higher disability [90]. Specific speech subsystems are often affected, including deviations in articulation, prosody and respiratory support, and voice quality [91]. These deficits are linked to function of specific areas of the cerebellum and connected regions of the cerebrum (Fig. 2a and b) and are associated with other measures of disease severity in MS. For example, an increase in neurofilament light (NfL) levels following symptom onset correlate with dysarthria severity [92]. NfL is a protein associated with myelinated axons that is found in cerebrospinal fluid in amounts proportional to the level of axonal damage [93]. Speech profiles also change in line with performance on the 9HPT [94] and EDSS [91].

## Speech as a Potential Marker of Cerebellar Dysfunction in MS

Speech has shown potential as a clinical marker of disease in several other progressive neurological conditions, especially those with cerebellar involvement. These include Huntington’s disease [95], Friedreich’s ataxia (FA) [96, 97], and Parkinson’s disease (PD) [98, 99]. This research, along with recent studies regarding motor speech dysfunction in pwMS [88, 91] and people with cerebellar ataxia [100, 101], highlight the value of objective measures of dysarthria for monitoring MS disease progression associated with cerebellar dysfunction. While perceptual analysis (clinical listening and rating) of speech is the most used method for speech evaluation in clinical settings, it poses limitations such as low reproducibility, subjectivity, and confirmation bias [102, 103]. Acoustic speech analysis has been suggested to overcome the limitations of perceptual assessment [94, 103, 104]. It provides objective data linking the level of disability and speech impairment, suggesting that it is a suitable measure of MS-related neurological impairment [90]. Furthermore, acoustic speech analysis shows promise in detecting subclinical dysarthria in pwMS [91, 94, 104]. However, Noffs et al. [90] note that longitudinal research is required to determine whether acoustic speech analysis can be used as a marker for progression in pwMS.

As with any new potential biomarker, it is crucial to ensure that associations with speech variables and associations with MS function are relevant. This is determined by ecological validity, which must integrate any proposed translation of research tools into clinical use. To improve ecological validity of speech metrics, for instance, continuous or spontaneous speech should be analysed in addition to purposefully created speech tasks, such as sustained vowels or syllable repetition [105]. In doing this, the results will be more representative of natural speech and will therefore be more generalisable.

### Measuring the Subsystems of Speech

Impaired motor control and weakness of muscles involved in speech can lead to dysarthria, impacting all speech subsystems: respiration, articulation, phonation, prosody, and resonance [87]. Persons with cerebellar dysarthria often have reduced articulatory accuracy and slower articulation [87, 106], which can be measured using a combination of electroglottography (monitoring the vibration of the vocal folds) and acoustic speech analysis [106]. Articulation rate specifically could be used as a marker of progression in MS [107]. Furthermore, spectral and cepstral analysis focuses on the spectral representation of speech and can pinpoint the location of formants, which can be defined as the concentration of acoustic energy around peak frequencies in speech. Cepstral analysis has been used to monitor abnormalities in resonance and voice quality due to cerebellar ataxia [100] and dysarthria in persons with PD [108] and cerebral palsy [109]. Composite acoustic measures involving prosodic features (such as intonation, stress rhythm), feature selection, and support vector machines (SVMs) and dysarthria severity diagnosis can accurately select dysarthric features and predict diagnosis [110]. Additionally, combining SVM with a Gaussian mixture model (GMM) to develop an objective measure of dysarthria severity can be used to assess prosody [111]. There are also systems specifically for dysarthric speech, which use SVMs and hidden Markov models (HMMs) to increase the level of speech recognition [112]. Automatic speech recognition (ASR) systems, which are trained on databases of healthy speech, can be used to estimate the level of intelligibility of pathological speech [113]. “Dysarthria phenotyping” may also be achievable. As different causes of dysarthria can produce different speech recognition errors, ASR can differentiate between pwMS and people with FA, for example, by identifying dysarthric speech patterns [114].

### Acoustic Speech Analysis in pwMS

Objective methods of speech and voice analysis have allowed for better detection and characterisation of speech changes caused by MS [86, 94, 115]. Acoustic analysis can enhance our understanding of how neuromotor dysfunction in MS affects speech and for detecting and monitoring changes resulting from disease progression or treatment [86]. Research thus far has indicated that acoustic analysis of speech can produce valuable metrics related to neurological status in pwMS. See Table 2 for a summary of the current findings.

**Table 2:** Summary of research on acoustic speech analysis and cerebellar dysfunction

Author	Disorder	Number of participants	Method/design	Key findings
Hartelius, Buder [136]	MS	20 pwMS, 20 age- and gender-matched controls	Total variance, a magnitude-based analysis, and a frequency-based analysis were used to assess long-term phonatory instability in pwMS. Phonatory instability was measured both in Hz (frequency) and dB (sound intensity/loudness)	Instability of sound intensity can be used to discriminate between pwMS and healthy controls
Jannetts and Lowit [108]	PD, ataxia	43 pwPD, ten pw ataxia	Sustained phonation of /a/, passage reading, and spontaneous speech was recorded and analysed using acoustic measures and perceptual analysis	Cepstral peak prominence is an adequate predictor of breathiness and dysphonia in persons with motor speech disorders
Kuo and Tjaden [137]	MS, PD	15 pwMS, 12 pwPD, 14 controls	Participants were recorded reading a 192-word passage three times in a cross-sectional design. The passage was read normally, loudly, and slowly. The latter two conditions’ orders were counterbalanced and randomised	Passage reading is associated with naturally occurring acoustic variation both in pwMS with dysarthria and in controls
Novotný, Rusz, Spálenka, Klempír, Horáková and Ruzicka [138]	MS, Multiple system atrophy, Cerebellar ataxia	74	Analyse nasality of speech in people with cerebellar disorders causing ataxic dysarthria using 1/3 octave spectra method	There can be abnormal fluctuations in nasality in pwMS with ataxic dysarthria. This was more prominent than differences in nasality between control and cerebellar disorder groups
Noffs, Perera, Kolbe, Shanahan, Boonstra, Evans, Butzkueven, van der Walt and Vogel [91]	MS	-	A systematic review of literature	Acoustic measurement of vowel instability can be used to discriminate between pwMS and controls. An increase in pausing, slower maximum speech rate, and subclinical voice tremor are predictive of cerebellar dysfunction in pwMS
Rusz, Tykalová, Salerno, Bancone, Scarpelli and Pellecchia [139]	MSA, PD	40 with probable MSA, 20pwPD, 20 controls	Use quantitative acoustic analysis to distinguish between MSA, PD, and controls	Speech disorders reflect underlying pathophysiology of MSA. Acoustic speech analysis can distinguish between people with MSA and PD due to differing dysarthric features
Kashyap, Pathirana [100]	Cerebellar ataxia	42 pwCA, 23 age-matched controls	A composite cepstral analysis comprising 12 measures was used to distinguish between people with cerebellar ataxia and control participants	Phase-based and magnitude-based cepstral analysis of speech performs were better than more traditional, time-based acoustic analysis in terms of discrimination between patients and controls
Noffs, Boonstra, Perera, Butzkueven, Kolbe, Maldonado, et al. [90]	MS	119 pwMS (68 completed MRI), 22 controls	Acoustic speech analysis assessed timing, control, voice quality, naturalness, and intelligibility. Additional perceptual analysis was used for comparison. PwMS also completed the EDSS and MS Impact Scale to assess quality of life. T1-weighted MRI was used to measure brain volume and lesion load	Composite speech scores correlate with disease severity, quality of life, and total lesion load. Measures of pause percentage and frequency instability correlate with EDSS scores in pwMS, even with no perceivable dysarthria. The perceptual analysis only picked up speech impairment in pwMS with established neurological impairment (EDSS ≥ 3)

## Electronic and Home-Based Monitoring Systems

Electronic and home-based services can improve healthcare by removing accessibility constraints. Digital healthcare, in particular self-monitoring tools, can provide patients with real-time feedback, increased support, awareness of their current status, and a sense of control over their disease [116, 117]. Those are particularly appealing attributes for long-term monitoring of symptoms [118]. Many tools have been developed to monitor and assess cerebellar MS symptoms outside clinical settings, including smartphone applications and activity trackers [116]. There is also the patient determined disease steps (PDDS), which is a measure of disability in MS that can be administered online. The PDDS significantly correlates with the EDSS, pyramidal, and cerebellar functional systems scores [119], making it a useful tool for monitoring cerebellar dysfunction-based disability in pwMS.

Floodlight is a smartphone app-based system for monitoring MS disease management and progression. This tool includes a collection of tasks designed to assess mood, information processing, hand motor function, gait, and balance [120, 121]. Both the pinching test and the draw a shape test included in Floodlight correlate significantly with the 9HPT [122]. Furthermore, smartphone-based versions of the U-turn speed test and 2-min walk test also included in Floodlight have been determined as reliable and valid measures of gait and balance in pwMS [123, 124]. Recently, there has also been interest in using speech as a biomarker of neurodegenerative diseases such as MS and PD, as well as for mental health, cardiovascular diseases and COVID-19 [125]. Speech data collection is flexible and can be done in clinic, and also at home via telephone, smartphone, and web-based recording systems [125]. Tablet-based analysis of acoustic speech measures shows promise for diagnosis, monitoring, and risk prediction in pwMS [126, 127]. However, it is essential to note that digital and home-based interventions tend to have low usage and high dropout rates [128] especially outside research situations. Patients are more likely to use digital monitoring consistently if the system tracks progress, are personalised and targeted, can adapt to changing needs, provide self-management techniques, and, most importantly, have the additional support of a clinician [129]. In using digital systems, there is the additional consideration of device issues that may impact the continued monitoring of symptoms. For example, different mobile devices may have different technical issues with the same health application. Furthermore, with at-home monitoring, assistance with technical issues is less readily available than in the clinic. Given this is a more recent area of MS research and management, there is not much validation data, but home-based measures show promise.

## Concluding Statements

Current tools for monitoring cerebellar dysfunction in pwMS present major limitations for both the detection of subclinical progression in cerebellar dysfunction and long-term tracking of disease progression. Cerebellar symptoms are associated with faster disease progression and earlier onset of SPMS, as well as reduced relapse recovery. For optimal monitoring of MS, we suggest working to determine the best combination of available measures for the patient. Furthermore, we must continue to explore new ways of assessing cerebellar dysfunction in pwMS. Conventional tools such as clinical tests and structural MRI are crucial for diagnosis, understanding MS and supporting pwMS with cerebellar dysfunction. However, they often do not meet our needs as researchers, or the disease management needs of the patients. While advanced neuroimaging provides additional information regarding functional and microstructural changes in the cerebellum, it is not without its limitations, and, like all imaging, it is not accessible to all pwMS due to cost, travel, and comfort. The step into digital and home-based monitoring has given rise to wearable monitors and smartphone applications to assess cerebellar symptoms such as gait and balance disruptions. In turn, this has provided pwMS with more accessible management and monitoring of their disease. Speech data is ideal for digital monitoring due to its ease of collection and ability to provide objective results in real time. The research to date suggests that speech would make a good marker of cerebellar dysfunction in pwMS. However, further research is required, particularly in terms of using acoustic speech analysis to monitor cerebellar changes over time associated with MS disease progression.
